# Evaluation of the Relationship Between Pain and Functional Status, Depression, Anxiety and Quality of Life in Patients with Spinal Cord Injury: Neuropathic Pain in Spinal Cord Injury

**DOI:** 10.3390/medicina61112047

**Published:** 2025-11-17

**Authors:** Zeynel Abidin Akar, Mehmet Karakoç, Mustafa Akif Sarıyıldız, Öznur Batmaz, Remzi Çevik

**Affiliations:** 1 Division of Rheumatology, Department of Physical Medicine and Rehabilitation, School of Medicine, Dicle University, 21280 Diyarbakır, Turkey; 2 Department of Physical Medicine and Rehabilitation, School of Medicine, Dicle University, 21280 Diyarbakır, Turkey; 3 Department of Physical Medicine and Rehabilitation, Memorial Dicle Hospital, 21220 Diyarbakır, Turkey; 4 Family Medicine, Ministry of Health 37th Family Health Center, 21070 Diyarbakır, Turkey

**Keywords:** spinal cord injury, neuropathic pain, quality of life, anxiety, depression, functional outcomes, LANSS

## Abstract

*Background and Objectives:* Neuropathic pain is a prevalent and disabling consequence of spinal cord injury (SCI), adversely affecting physical function, psychological health, social engagement, and overall quality of life. *Objectives*: This study aimed to determine the prevalence of neuropathic pain in patients with spinal cord injury (SCI) and to examine its associations with clinical and demographic factors, quality of life, depression, and anxiety. *Materials and Methods:* Eighty-four patients with spinal cord injury (SCI) who were admitted to the Department of Physical Medicine and Rehabilitation and followed up at the rehabilitation outpatient clinic of Dicle University Faculty of Medicine (Diyarbakır, Turkey) were included in the study. Neurological status was assessed using the American Spinal Injury Association (ASIA) scale. Functional ambulation was evaluated with the Walking Index for Spinal Cord Injury (WISCI) and the Functional Ambulation Scale (FAS), while independence was measured using the Spinal Cord Independence Measure, version 3 (SCIM-III). Quality of life was assessed with the Short Form-36 (SF-36), and depression and anxiety were evaluated using the Beck Depression Inventory (BDI) and Beck Anxiety Inventory (BAI), respectively. The severity of neuropathic pain, fatigue, and paresthesia was assessed using the Visual Analog Scale (VAS) and the Leeds Assessment of Neuropathic Symptoms and Signs (LANSS) questionnaire. *Results*: Neuropathic pain was observed in 41.7% of patients. No significant differences were found in age, sex, or marital status between patients with and without neuropathic pain. Patients with neuropathic pain had significantly higher Beck Anxiety Inventory (BAI) scores and lower scores in several Short Form-36 (SF-36) domains, including vitality, bodily pain, and emotional well-being. Leeds Assessment of Neuropathic Symptoms and Signs (LANSS) scores were positively correlated with Visual Analog Scale (VAS) fatigue and BAI scores, and negatively correlated with SF-36 domains such as vitality, general health, and bodily pain. *Conclusions*: Neuropathic pain is a common and debilitating complication following spinal cord injury (SCI). It is closely associated with reduced quality of life and heightened psychological distress, particularly anxiety. Early recognition and effective management of neuropathic pain are crucial for optimizing functional recovery and enhancing psychosocial well-being in patients with SCI.

## 1. Introduction

Spinal cord injury (SCI) is a debilitating, life-altering condition that not only impairs motor and sensory functions but also profoundly affects psychological well-being, autonomy, and social relationships. The consequences of SCI extend beyond physical impairments, influencing personality, emotional stability, and family and community dynamics. In addition to these psychosocial burdens, SCI presents complex medical challenges that necessitate long-term, multidisciplinary management. These challenges may include chronic pain, spasticity, pressure ulcers, urinary and bowel dysfunction, and an increased risk of infections.

Despite these challenges, significant advancements in medical care, rehabilitation techniques, and assistive technologies over the past decades have improved functional outcomes, enhanced quality of life, and increased life expectancy among individuals with spinal cord injury (SCI) [1,2]. Consequently, there is a growing emphasis not only on survival but also on optimizing physical function, mental health, and social integration in this population.

Following spinal cord injury (SCI), one of the most immediate and profound consequences is the loss of motor function. Neuropathic pain (NP), however, is a common and particularly distressing complication that can develop acutely or chronically after injury. This type of pain may significantly impair functional capacity, psychological well-being, and overall quality of life. Post-SCI pain has diverse etiologies and clinical manifestations, appearing either immediately after injury or months to years later. Despite its high prevalence and substantial impact, there is no universal consensus among researchers and clinicians regarding the classification, diagnosis, or prevalence of the various pain types observed in SCI populations [3,4].

Chronic pain is highly prevalent among individuals with spinal cord injury (SCI), with reported rates ranging from 11% to 94%, depending on study populations and diagnostic criteria [5,6]. Approximately 30% of individuals experiencing chronic pain are diagnosed with neuropathic pain [5]. According to the Spinal Cord Injury Pain Task Force of the International Association for the Study of Pain (IASP), pain in SCI patients is broadly classified into two main categories: nociceptive and neuropathic pain [7]. Unlike nociceptive pain, neuropathic pain does not result from external tissue injury but arises from pathological processes within the central or peripheral nervous system as a consequence of the lesion itself [8].

Neuropathic pain (NP) profoundly affects multiple aspects of an individual’s well-being. It is frequently associated with mood disturbances, heightened anxiety, and sleep disruption, all of which can intensify the subjective experience of pain. Sleep disturbances and depressive symptoms often interfere with the ability to perform activities of daily living, contributing to greater disability [9,10]. Beyond the direct consequences of motor impairment, these psychological and behavioral factors further limit participation in rehabilitation programs and delay reintegration into work or other productive roles. This highlights the complex, bidirectional relationship between pain, mental health, and functional outcomes in individuals with spinal cord injury (SCI) experiencing neuropathic pain. Overall, neuropathic pain in SCI represents a significant clinical challenge, restricting functional independence and substantially impairing quality of life [11].

The primary objective of this study was to determine the prevalence of neuropathic pain among individuals with spinal cord injury (SCI) and to examine its associations with demographic and clinical characteristics. Additionally, we aimed to investigate the relationships between neuropathic pain, functional status, quality of life, and psychological factors, including anxiety and depression, in this population. Through this comprehensive approach, we sought to enhance understanding of the multifaceted impact of neuropathic pain in SCI and to provide insights that may inform the development of more effective, targeted management strategies.

## 2. Materials and Methods

### 2.1. Study Design and Study Population

This prospective observational cross-sectional study was conducted in the Department of Physical Medicine and Rehabilitation at Dicle University Faculty of Medicine between November 2015 and November 2016. During this period, 120 patients with a confirmed diagnosis of spinal cord injury (SCI) who attended the rehabilitation outpatient clinic were prospectively evaluated for eligibility. Adult patients (≥18 years) capable of completing all required assessments were included. Exclusion criteria comprised lack of consent (*n* = 10), incomplete medical records (*n* = 10), and other predefined criteria (*n* = 16). After applying these criteria, 84 patients were enrolled and included in the final analyses.

All included participants were either admitted to the inpatient rehabilitation unit or followed up in the rehabilitation outpatient clinic. Informed consent was obtained from all participants prior to inclusion. The study was conducted in accordance with the principles of the Declaration of Helsinki and was approved by the Institutional Review Board of Dicle University Faculty of Medicine (Protocol Code: 430; approval date: 23 October 2015).

**Figure 1:**
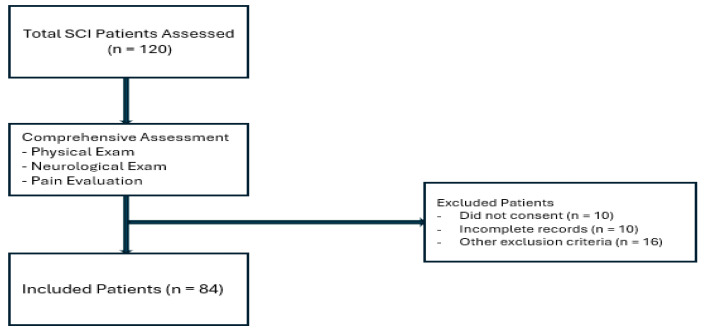
Study population and assessment for spinal cord injury patients.

The flowchart illustrates the study population selection and data collection process. A total of 120 spinal cord injury patients were initially assessed. Patients were excluded if they did not provide consent (*n* = 10), had incomplete records (*n* = 10), or met other exclusion criteria (*n* = 16). The remaining 84 patients were included in the study, provided informed consent, and underwent a comprehensive assessment including physical and neurological examination as well as pain evaluation. Demographic and clinical data were collected using a structured questionnaire. The recruitment process and participant flow are summarized in Figure 1.

Neurological status was assessed using the American Spinal Injury Association (ASIA) Impairment Scale, which enables a detailed evaluation of both sensory and motor function [12]. Functional ambulation was evaluated with the Walking Index for Spinal Cord Injury (WISCI), and overall functional independence was measured using the Spinal Cord Independence Measure, version III (SCIM-III) [13,14]. These standardized instruments provided a comprehensive characterization of participants’ neurological deficits, functional capacities, and levels of independence following SCI.

Quality of life was assessed using the Short Form-36 (SF-36) questionnaire, which evaluates eight domains: Physical Functioning, Role-Physical, Bodily Pain, General Health, Vitality, Social Functioning, Role-Emotional, and Mental Health. Each domain is scored from 0 to 100, with higher values reflecting better perceived health status [15]. A total SF-36 score was calculated as the mean of all domain scores to provide an overall measure of quality of life. In addition, the Physical Component Summary (PCS) and Mental Component Summary (MCS) scores were computed according to the standard SF-36 scoring algorithm, representing the physical and mental dimensions of health-related quality of life, respectively.

The Beck Depression Inventory (BDI) and Beck Anxiety Inventory (BAI) were administered to assess the presence and severity of depressive and anxiety symptoms [16,17]. Pain assessment was performed for all participants, and the diagnosis of neuropathic pain was established based on a detailed medical history and clinical examination. Participants were subsequently categorized into two groups: those with neuropathic pain and those without. In the neuropathic pain group, the Leeds Assessment of Neuropathic Symptoms and Signs (LANSS) was used to further characterize pain features [18], while pain intensity was quantified using the Visual Analog Scale (VAS) [19].

Neuropathic pain was assessed using the Leeds Assessment of Neuropathic Symptoms and Signs (LANSS) pain scale, which includes both symptom-based and sensory examination components. A LANSS score ≥12 was considered indicative of neuropathic pain, in accordance with established validation studies. In addition to diagnostic confirmation, the total LANSS score was also used to quantify neuropathic pain severity. The Turkish version of the LANSS, which has been previously validated for reliability and accuracy, was used in this study [20].

Pain intensity was evaluated using a Visual Analog Scale (VAS) ranging from 0 (“no pain”) to 10 (“worst imaginable pain”), based on the patients’ average pain intensity during the previous week. Separate VAS ratings were obtained for spontaneous pain, paresthesia, and fatigue when applicable.

To maintain a homogeneous and focused study population, several exclusion criteria were applied. Patients younger than 16 years, those with cognitive impairment, or those unable to cooperate with study procedures were excluded. Additionally, individuals with pre-existing organ or peripheral nerve injuries, unstable clinical conditions, or comorbidities such as diabetes mellitus that could independently contribute to neuropathic pain were not included. Patients in the acute phase of spinal cord injury or experiencing spinal shock were also excluded. These criteria were designed to minimize potential confounding factors and enhance the internal validity of the study, thereby improving the accuracy and reliability of the findings.

### 2.2. Sample Size and Power

Since this was a single-center prospective observational study, no a priori sample size calculation was performed. A post hoc power analysis based on the observed data (*n* = 84) showed that the study had 83–99% power to detect the observed correlations (*r* = 0.32–0.54) and approximately 92% power for the multiple regression model (*R*^2^ = 0.41, *f*^2^ = 0.23, α = 0.05). These results indicate that the sample size was adequate to detect moderate-to-large effect sizes.

### 2.3. Statistical Analysis

Statistical analyses were performed using SPSS version 27.0 (IBM Corp., Armonk, NY, USA). The normality of data distribution was assessed using the Shapiro–Wilk test, along with visual inspection of histograms and Q–Q plots. Normally distributed variables were presented as mean ± standard deviation (SD), whereas non-normally distributed variables were expressed as median (interquartile range, IQR). Depending on data distribution, either the independent-samples *t*-test or the Mann–Whitney U test was applied for group comparisons. Correlations between continuous variables were evaluated using Pearson or Spearman correlation coefficients, as appropriate.

To account for multiple comparisons in SF-36 domain and correlation analyses, Bonferroni correction was applied to control the type I error rate. Effect sizes were reported as Cohen’s d for group comparisons and standardized β coefficients or correlation coefficients (*r*) for regression and correlation analyses. Ninety-five percent confidence intervals (95% CI) were provided for key estimates, and a two-tailed *p* value of <0.05 was considered statistically significant. Multivariate analyses were conducted using multiple linear regression to identify independent predictors of neuropathic pain severity. The results of the multiple linear regression analysis, including all tested variables, are shown in Supplementary Table S1.

## 3. Results

A total of 84 patients with spinal cord injury (SCI) were included in the study. Of these, 56 (66.7%) were male. The mean age of participants was 36.8 ± 14.4 years (range: 16–70 years), and the mean disease duration was 30 months (range: 6–480 months). Regarding marital status, 53 patients (63.1%) were married (Table 1).

Educational status varied among the participants: 17 patients (20.2%) were illiterate, 22 (26.2%) had completed primary school, 14 (16.7%) secondary school, 29 (34.5%) high school, and 2 (2.4%) held a university degree. Regarding employment status, only 6 patients (7.1%) were actively employed, whereas 78 (92.9%) were unemployed at the time of assessment.

The etiology of spinal cord injury (SCI) was traumatic in 56 patients (66.7%) and non-traumatic in 28 patients (33.3%). Among traumatic injuries, falls from height were the most common cause (28 patients, 33.3%), followed by traffic accidents (14 patients, 16.7%), and iatrogenic causes (8 patients, 10.0%). Non-traumatic injuries were attributed to medical or disease-related conditions. According to the American Spinal Injury Association (ASIA) classification, 30 patients (35.7%) had sensory complete injuries, whereas 54 (64.3%) had sensory incomplete lesions. In terms of motor function, 10 patients (11.9%) were classified as tetraplegic, while the majority, 74 patients (88.1%), were paraplegic.

These demographic and clinical characteristics provide a comprehensive overview of the study population, highlighting the heterogeneity in etiology, neurological impairment, and socioeconomic background among individuals with spinal cord injury (SCI).

Among the study participants, 26 patients (31%) exhibited spasticity, characterized by involuntary muscle contractions commonly observed following spinal cord injury (SCI). In addition, pressure ulcers were present in 12 patients (14.3%), representing localized tissue damage resulting from sustained pressure on the skin and underlying structures. These findings provide further descriptive insight into the clinical characteristics of the study population.

The patients’ functional and psychological assessment scores—including the Walking Index for Spinal Cord Injury (WISCI), Spinal Cord Independence Measure version 3 (SCIM-III), Short Form-36 Health Survey (SF-36), Beck Depression Inventory (BDI), and Beck Anxiety Inventory (BAI)—are summarized in Table 1.

Neuropathic pain was reported in 35 patients (41.7%), whereas 49 patients (58.3%) did not report neuropathic pain. Among those experiencing neuropathic pain, 74.3% were male and 25.7% were female, compared with 61.2% males and 38.8% females in the painless group. Regarding marital status, 71.4% of patients with neuropathic pain were married, compared with 57.1% in the group without pain. No statistically significant differences were observed between the groups in terms of age, gender, or marital status (*p* > 0.05) (Table 2).

Comparison of educational status between the groups revealed no statistically significant differences. The mean disease duration was 24 months (range: 6–480 months) in the neuropathic pain group and 16 months (range: 6–480 months) in the painless group, with no significant difference observed between the two groups (*p* > 0.05).

No significant differences were observed between the groups regarding neurological level or etiology (traumatic versus non-traumatic causes). Among patients with neuropathic pain, 14 (40%) were classified as having complete SCI according to the ASIA scale, while 21 (60%) had incomplete SCI. In the painless group, 16 patients (32.7%) had complete SCI and 33 (67.3%) had incomplete SCI. In terms of motor function, 4 patients (11.4%) with neuropathic pain were tetraplegic, and 31 (88.6%) were paraplegic. Similarly, in the painless group, 6 patients (12.2%) were tetraplegic, and 43 (87.8%) were paraplegic. Statistical analysis revealed no significant differences between the groups in terms of neurological level or completeness of injury (*p* > 0.05).

The mean Beck Depression Inventory (BDI), Beck Anxiety Inventory (BAI), and Short Form-36 (SF-36) scores for the patient groups are summarized and compared in Table 3. The mean Spinal Cord Independence Measure version 3 (SCIM-III) score was 63.2 ± 26.3 in patients with neuropathic pain and 67.91 ± 25.84 in the painless group, with no statistically significant difference observed (*p* > 0.05). Similarly, functional ambulation levels, assessed using the Walking Index for Spinal Cord Injury (WISCI), were 10.97 ± 7.6 and 11.75 ± 7.47 in the neuropathic pain and painless groups, respectively, with no significant difference detected (*p* > 0.05). These findings provide a descriptive comparison of functional and psychological outcomes between patients with and without neuropathic pain.

When comparing psychological parameters and quality of life, patients in the neuropathic pain group had a higher mean BDI score (22.28 ± 11.61) than those in the painless group (18.81 ± 10.90). Similarly, the mean BAI score was higher in the neuropathic pain group (18.17 ± 10.30) compared with the painless group (14.42 ± 9.42). In contrast, overall quality of life, as measured by the total SF-36 score, was lower among patients with neuropathic pain (33.14 ± 18.70) than in those without (45.82 ± 21.73). Statistically significant differences were observed in the SF-36 domains of vitality, emotional well-being, bodily pain, and total quality of life score (*p* < 0.05). These results provide a descriptive comparison, suggesting that patients with neuropathic pain experience higher levels of psychological distress—particularly anxiety—and lower quality of life relative to those without neuropathic pain (Table 3).

Correlation analyses between the Leeds Assessment of Neuropathic Symptoms and Signs (LANSS) pain score and clinical variables—including age, BDI, BAI, ASIA scale, SCIM-III, WISCI, SF-36 domains, and Visual Analog Scale (VAS) scores for pain and fatigue—are summarized in Table 4. The LANSS score demonstrated a significant positive correlation with BAI scores (*r* = 0.323, *p* = 0.003), indicating an association between higher anxiety levels and greater neuropathic pain severity. Conversely, significant negative correlations were observed between LANSS scores and several SF-36 domains, including total SF-36 score (*r* = −0.544, *p* < 0.001), bodily pain (*r* = −0.544, *p* < 0.001), vitality (*r* = −0.351, *p* = 0.001), social functioning (*r* = −0.259, *p* = 0.017), role emotional (*r* = −0.286, *p* = 0.008), and mental health (*r* = −0.299, *p* = 0.006). No significant correlations were observed between LANSS scores and BDI (*r* = 0.188, *p* = 0.087), SCIM-III (*r* = 0.111, *p* = 0.316), ASIA scale (*r* = 0.166, *p* = 0.132), WISCI score (*r* = 0.118, *p* = 0.283), or age (*r* = 0.137, *p* = 0.214).

Taken together, these findings indicate that higher neuropathic pain severity is associated with increased anxiety and lower quality of life across multiple domains, while no significant associations were observed with depression, neurological impairment, functional independence, ambulation capacity, or age.

The mean VAS pain score in the neuropathic pain group was 6.94 ± 2.09, indicating moderate pain intensity, and was significantly higher than that of the painless group (*p* = 0.001). Similarly, the mean VAS fatigue score was 7.05 ± 2.20 in the neuropathic pain group, reflecting moderate severity, and differed significantly from the painless group (*p* = 0.027). The mean VAS paresthesia score was 8.05 ± 1.57 in the neuropathic pain group, indicating a relatively high level of paresthesia, which was significantly greater than in the painless group (*p* < 0.001) (Table 4). Regarding the character of neuropathic pain, 33 patients (39.3%) reported experiencing a “burning” sensation.

A multiple linear regression analysis was performed to identify independent predictors of neuropathic pain severity, as measured by the LANSS pain score, in patients with spinal cord injury. The overall model was statistically significant (F(18,65) = 7.46, *p* < 0.001) and explained 58.4% of the variance in LANSS scores (adjusted R^2^ = 0.584). Among the variables entered into the model, VAS Paresthesia, SCIM-III score, and SF-36 General Health emerged as significant independent predictors. Higher paresthesia intensity and better perceived general health were associated with increased LANSS pain scores, which may reflect greater awareness and reporting of neuropathic symptoms among patients with better overall health. In contrast, greater functional independence (higher SCIM-III scores) was associated with lower pain severity.

These findings, summarized in Table 5, underscore the multifactorial nature of neuropathic pain in spinal cord injury and emphasize the interrelated roles of sensory, functional, and psychosocial factors. Understanding these relationships may assist clinicians in developing more individualized, multidimensional pain management strategies aimed at improving quality of life in this population.

## 4. Discussion

Neuropathic pain in individuals with spinal cord injury (SCI) is a complex and challenging condition that substantially affects patients’ quality of life and daily functioning. Unlike nociceptive pain, which originates from tissue damage or inflammation, neuropathic pain results from injury or dysfunction within the nervous system. In SCI patients, it often presents as burning, tingling, shooting, or electric shock-like sensations, typically occurring below the level of injury and sometimes radiating to the extremities. The severity and duration of symptoms are highly variable, with some patients experiencing persistent pain and others reporting intermittent episodes [21].

The prevalence of neuropathic pain in our cohort (41.7%) is consistent with rates reported in previous studies. Siddall et al. observed a similar prevalence of approximately 40% among individuals with chronic SCI [22]. Similarly, a systematic review by Finnerup et al. reported a prevalence range of 11% to 96%, reflecting substantial heterogeneity across studies due to differences in study design, patient characteristics, and pain assessment methods [23]. Despite these variations, the evidence consistently indicates that neuropathic pain is a common and clinically significant issue among individuals with SCI.

Consistent with our results, several previous studies have reported no significant associations between neuropathic pain and demographic variables such as age, sex, or marital status [22,24,25]. These findings suggest that the occurrence of neuropathic pain in individuals with SCI is not strongly related to demographic characteristics. Nonetheless, pain perception is inherently subjective and influenced by a complex interplay of individual, psychological, and social factors, which may contribute to the variability observed across studies.

Regarding disease-related factors, no significant difference in SCI duration was observed between patients with and without neuropathic pain. This observation aligns with previous studies reporting no clear association between time since injury and the presence of neuropathic pain [25]. However, some studies have suggested that the chronicity of SCI may be related to the onset and persistence of neuropathic pain [26]. These findings highlight the need for further longitudinal studies with larger cohorts to better characterize the relationship between disease duration and neuropathic pain in individuals with SCI.

The etiology of spinal cord injury (SCI) has been examined as a potential factor associated with neuropathic pain. In the present study, no significant difference in neuropathic pain prevalence was observed between traumatic and non-traumatic causes. This observation is consistent with previous studies reporting no clear association between the underlying cause of SCI and the occurrence of neuropathic pain [22,24,25]. The pathophysiology of post-SCI neuropathic pain is multifactorial and complex, involving both peripheral and central sensitization processes. Therefore, the etiology of the injury itself may have a limited influence compared with neurophysiological mechanisms in relation to the presence of neuropathic pain.

Regarding neurological classification, our findings showed no significant differences in injury completeness or neurological level between patients with and without neuropathic pain. These observations align with previous systematic reviews indicating that neuropathic pain is not strongly associated with the severity or level of SCI [26]. Nevertheless, individual variations in pain processing and the involvement of distinct sensory pathways may contribute to the presentation of neuropathic pain, independent of the anatomical level or completeness of the lesion.

Our findings further highlight the impact of neuropathic pain on psychological well-being and overall quality of life in individuals with SCI. Patients with neuropathic pain exhibited higher levels of depression and anxiety, along with lower scores across several domains of the SF-36 health survey. These observations are consistent with previous research demonstrating the association between neuropathic pain and adverse psychological and functional outcomes in this population [22,25,26]. Chronic pain may be related to increased emotional distress, social withdrawal, and limitations in daily functioning, underscoring the value of multidisciplinary pain management strategies that address both physical and psychological aspects of care.

An important methodological consideration in neuropathic pain research is the choice of assessment instrument. In this study, the Leeds Assessment of Neuropathic Symptoms and Signs (LANSS) scale was employed, as it is a widely accepted and validated tool for evaluating neuropathic pain in SCI populations [23,26]. Other validated instruments—such as the Neuropathic Pain Scale (NPS) and the Douleur Neuropathique en 4 Questions (DN4)—may provide a more detailed and multidimensional assessment of neuropathic pain characteristics. Future studies could consider the concurrent use of multiple standardized tools to enhance the accuracy and comprehensiveness of neuropathic pain evaluation.

The results of this study demonstrated associations between the LANSS pain score and several psychological and quality-of-life measures in individuals with spinal cord injury (SCI). The LANSS pain score was positively correlated with the Beck Anxiety Inventory (BAI) score, indicating that higher levels of neuropathic pain co-occurred with elevated anxiety. This observation is consistent with previous research highlighting the interplay between pain and anxiety in individuals with SCI [27].

The LANSS pain score also demonstrated significant negative correlations with several domains of the SF-36 quality-of-life questionnaire, including bodily pain, vitality, social functioning, role emotional, and mental health. These observations suggest that higher neuropathic pain levels co-occur with lower overall quality of life and reduced functioning across both physical and psychosocial domains. This underscores the multidimensional nature of neuropathic pain, which extends beyond sensory disturbances to include effects on emotional well-being, social participation, and mental health. Consistent with these findings, previous studies have reported notable associations between neuropathic pain and various quality-of-life parameters [28,29].

No significant correlations were observed between the LANSS pain score and depression (as measured by the BDI), neurological injury level, functional independence (SCIM-III), ambulation status (WISCI), or age. These observations indicate that, within this cohort, neuropathic pain severity as assessed by the LANSS scale did not co-vary with these clinical or demographic characteristics. This is consistent with previous studies reporting similar findings [30,31]. Other factors—such as central sensitization, psychosocial influences, or individual pain coping mechanisms—may contribute to variations in neuropathic pain intensity.

Notably, LANSS scores were significantly correlated with anxiety levels (BAI) but not with depressive symptoms (BDI). This pattern indicates that anxiety may exert a more prominent influence on neuropathic pain perception in individuals with spinal cord injury. Anxiety is known to increase somatic vigilance and amplify pain sensitivity through central sensitization mechanisms [32], whereas depressive symptoms may affect pain via more chronic and generalized affective pathways [33]. In the early or subacute phases following injury, anxiety-related hyperarousal and uncertainty regarding functional recovery may therefore exert a stronger impact on pain experience than depressive mood [34]. These observations are consistent with previous studies suggesting that anxiety can mediate the relationship between neuropathic pain and quality of life more strongly than depression in this population [27]. Further longitudinal investigations are needed to clarify these differential pathways and to inform targeted psychological and pain management interventions in SCI patients.

In the multiple linear regression analysis, paresthesia intensity (VAS paresthesia), functional independence (SCIM-III), and perceived general health (SF-36 General Health) emerged as significant independent predictors of neuropathic pain severity. The positive association between paresthesia intensity and LANSS scores is consistent with previous studies demonstrating that sensory disturbances such as tingling, burning, or electric-like sensations are core components of neuropathic pain following spinal cord injury (SCI) [35,36]. Notably, higher perceived general health was also associated with greater pain severity. This seemingly paradoxical relationship may reflect that individuals with better overall health and heightened bodily awareness are more likely to detect and report neuropathic symptoms, as previously discussed by Siddall et al. and Widerström-Noga et al. [22,28]. Conversely, greater functional independence, as indicated by higher SCIM-III scores, was associated with lower LANSS scores. This finding supports the notion that enhanced mobility, self-care, and social participation may mitigate pain perception through mechanisms such as improved neuroplastic adaptation, better coping strategies, or reduced psychosocial distress [37,38].

Taken together, these results underscore the complex interplay between sensory, functional, and psychosocial dimensions in shaping the experience of neuropathic pain after SCI. Recognizing these multidimensional influences is essential for designing individualized rehabilitation and pain management strategies that address not only nociceptive mechanisms but also functional capacity and psychological well-being.

The Visual Analog Scale (VAS) assessments for pain and fatigue provide insight into the subjective symptom burden experienced by individuals with neuropathic pain following SCI. VAS pain and fatigue scores were higher in the neuropathic pain group compared with the painless group, indicating a greater symptom burden in this population. The moderate pain intensity reported by patients with neuropathic pain suggests that pain is not only prevalent but also clinically relevant, consistent with previous studies documenting high prevalence and severity of neuropathic pain in individuals with SCI [22,26]. These observations underscore the importance of comprehensive pain management strategies that address both sensory-discriminative and affective-emotional dimensions to support patient outcomes.

The moderate levels of fatigue observed in the neuropathic pain group indicate that fatigue is a common and impactful symptom that may affect functional capacity and quality of life. Previous studies have reported an association between neuropathic pain and increased fatigue in individuals with SCI [22,39]. These observations highlight the importance of management strategies that consider both pain and fatigue in order to support rehabilitation outcomes, maintain energy levels, and optimize overall functional performance.

The differences observed in VAS pain and fatigue scores between groups highlight the clinical relevance of these symptoms. Healthcare providers may consider comprehensive assessment tools and multi-modal intervention strategies that combine pharmacological and non-pharmacological approaches. Pharmacological options can include analgesics and agents targeting neuropathic pain or fatigue, while non-pharmacological interventions—such as physical therapy, structured exercise programs, and cognitive-behavioral therapy—may support symptom management. Integrating these complementary strategies can contribute to improved functional outcomes and quality of life in individuals with SCI.

Furthermore, the subjective nature of pain and fatigue emphasizes the importance of a patient-centered approach in planning and implementing interventions. Regular, systematic assessments of pain and fatigue, combined with ongoing communication with patients, can support individualized treatment adjustments and help optimize clinical outcomes. By addressing both pain and fatigue in individuals with neuropathic pain, healthcare providers may contribute to improvements in overall well-being, functional capacity, and quality of life.

Regarding the management of neuropathic pain in individuals with SCI, although our study did not specifically investigate treatment strategies, it is important to note that effective management can be challenging due to the complex and multifactorial nature of neuropathic pain. Current therapeutic approaches often involve multimodal strategies, including pharmacological agents such as anticonvulsants, antidepressants, and opioids, alongside non-pharmacological interventions such as physical therapy, psychological support, and neurostimulation techniques [23,25].

There is growing interest in alternative and complementary therapies for managing neuropathic pain in individuals with SCI. Approaches such as transcutaneous electrical nerve stimulation (TENS), acupuncture, and cognitive-behavioral therapy (CBT) are increasingly being investigated. Although evidence regarding their efficacy in SCI-related neuropathic pain remains preliminary, some studies have reported promising findings [40,41]. Further research is needed to better characterize the effectiveness and potential long-term benefits of these interventions in this patient population.

An additional consideration is the impact of neuropathic pain on functional outcomes, including ambulation and independence in daily activities. In this study, no significant differences were observed in SCIM-III and WISCI scores between patients with and without neuropathic pain, suggesting that neuropathic pain did not co-vary with functional ability in this cohort. Nevertheless, pain may be associated with reduced motivation, limited participation in physical activities, and lower overall quality of life [42]. Further research is needed to clarify the interactions between neuropathic pain, functional status, and rehabilitation outcomes in this population.

These findings carry important implications for clinical rehabilitation in patients with spinal cord injury. The demonstrated link between neuropathic pain and anxiety emphasizes the value of routine psychological screening as part of a comprehensive patient assessment. Early identification of anxiety symptoms may help detect individuals at higher risk of poorer functional outcomes and diminished quality of life.

Incorporating psychological evaluation and interventions into neuropathic pain management protocols can enhance the overall effectiveness of rehabilitation programs. Multidisciplinary strategies that integrate pharmacological, physical, and behavioral approaches may yield synergistic benefits, targeting both the somatic and emotional components of pain. Individualized treatment plans that address anxiety and other psychosocial factors could improve adherence, patient satisfaction, and long-term functional recovery.

Collectively, these results highlight the importance of integrating mental health support into routine clinical care for spinal cord injury patients with neuropathic pain, reinforcing a holistic rehabilitation approach that encompasses both physical and psychological well-being.

In interpreting our findings, several important limitations should be acknowledged. First, the cross-sectional nature of the study precludes any inference of causality between neuropathic pain, psychological status, and quality of life. While these associations are consistent with previous reports linking neuropathic pain to depressive symptoms and impaired quality of life in individuals with spinal cord injury, longitudinal and prospective designs are necessary to elucidate the directionality and potential mediating mechanisms underlying these relationships. Such studies could help determine whether psychological distress exacerbates pain perception, or conversely, whether chronic neuropathic pain contributes to mood disturbances and reduced life satisfaction.

Second, although the inclusion and exclusion criteria were carefully designed to obtain a relatively homogeneous sample and reduce the influence of confounding variables, the exclusion of patients with diabetes mellitus and other neuropathic conditions may limit the generalizability of the findings. These conditions were excluded to minimize overlapping etiologies of neuropathic pain that could independently affect psychological or functional outcomes, thereby ensuring greater internal validity. However, given that comorbid metabolic and neurological disorders are relatively common in the spinal cord injury population, future studies involving more heterogeneous cohorts would provide a broader and more ecologically valid understanding of neuropathic pain and its psychosocial correlates.

Finally, the reliance on self-reported instruments may introduce response bias or subjective variability, although validated and widely used measures were employed. Incorporating objective assessments, such as neurophysiological or imaging-based correlates of neuropathic pain, could enhance the robustness of future research.

Despite these limitations, our study contributes to the growing body of evidence highlighting the multidimensional nature of neuropathic pain in individuals with spinal cord injury. By demonstrating significant associations between pain severity, psychological distress, and quality of life, our findings underscore the importance of a comprehensive biopsychosocial approach to pain assessment and management in this population.

## 5. Conclusions

In conclusion, this study adds to existing evidence on the high prevalence of neuropathic pain among individuals with spinal cord injury and its associations with mental health and quality of life. Despite advances in assessment and management strategies, there is a continued need to investigate alternative therapeutic approaches and to address the multidimensional aspects of neuropathic pain in relation to functional outcomes. Enhanced understanding of neuropathic pain in this population may inform the development of comprehensive, patient-centered care models aimed at supporting overall well-being and quality of life in individuals living with spinal cord injury.

## Figures and Tables

**Table 1:** Mean demographic, SCIM-III, BDI, BAI, and SF-36 scores of patients with spinal cord injury.

Parameter	Mean ± SD (Range) or *n* (%)
Age (years)	36.8 ± 14.4 (16–70)
Gender (Male/Female)	56/28
Marital status (Married/Single)	53/31
Disease duration (months)	Median 30 (6–480)
SCIM-III score	65.9 ± 26.0
BDI score	20.3 ± 11.3
BAI score	16.0 ± 9.9
SF-36 total score	40.5 ± 21.4

Abbreviations: SCIM-III = Spinal Cord Independence Measure version 3; BDI = Beck Depression Inventory; BAI = Beck Anxiety Inventory; SF-36 = Short Form-36 Health Survey.

**Table 2:** Demographic characteristics of patients with and without neuropathic pain.

Parameters	Neuropathic Pain Group (*n* = 35)	Painless Group (*n* = 49)	*p*-Value
Age (years, mean ± SD)	38.85 ± 13.30	35.26 ± 15.09	0.81
Gender (Male/Female)	26/9	30/19	0.247
Marital status (Married/Single)	25/10	28/21	0.054

Notes: Values are presented as mean ± standard deviation (SD) or number of patients. Statistical comparisons were performed using Student’s *t*-test for continuous variables and the Chi-square test for categorical variable.

**Table 3:** Comparison of BAI, BDI, and SF-36 scores between patients with and without neuropathic pain.

Parameters	Neuropathic Pain Group (*n* = 35)	Painless Group (*n* = 49)	*p*
General Health	39.4 ± 19.88	41.77 ± 24.16	0.634
Vitality	28.38 ± 25.80	40.10 ± 26.48	0.047
Social Functioning	37.54 ± 31.08	45.91 ± 35.84	0.269
Role Emotional	30.72 ± 40.26	64.34 ± 47.52	0.022
Mental Health	50.17 ± 23.28	60.06 ± 26.43	0.080
Physical Capacity	47.60 ± 48.56	55.10 ± 50.25	0.496
Bodily Pain	28.70 ± 19.46	51.53 ± 24.71	<0.001
Physical Functioning	3.14 ± 7.08	8.46 ± 16.58	0.078
Total SF-36 Score	33.14 ± 18.70	45.82 ± 21.73	0.007
BDI Score	22.28 ± 11.61	18.81 ± 10.90	0.166
BAI Score	18.17 ± 10.30	14.42 ± 9.42	0.048

Abbreviations: SF-36, Short Form-36 Health Survey; BDI, Beck Depression Inventory; BAI, Beck Anxiety Inventory. Statistically significant at *p* < 0.05; highly significant at *p* < 0.001.

**Table 4:** Correlation analysis between LANSS pain score and clinical parameters.

Parameters (*n* = 84)	*r*	*p*
SCIM 3	−0.111	0.316
BDI	0.188	0.087
BAI	0.323	0.003 **
SF 36 TOTAL	−0.421	0.000 **
SF 36 Vitality	−0.351	0.001 **
SF 36 Bodily pain	−0.544	0.000 **
SF 36 Social functioning	−0.259	0.017 *
SF 36 Emotional role	−0.286	0.008 **
SF 36 Mental health	−0.299	0.006 **
VAS Fatigue	0.323	0.003 **
Age	0.237	0.214
WISCI	0.118	0.283

Abbreviations: LANSS, Leeds Assessment of Neuropathic Symptoms and Signs; BDI, Beck Depression Inventory; BAI, Beck Anxiety Inventory; SF-36, Short Form-36 Health Survey; VAS, Visual Analogue Scale; SCIM-III, Spinal Cord Independence Measure III; WISCI, Walking Index for Spinal Cord Injury. * *p* < 0.05; ** *p* < 0.01.

**Table 5:** Significant Independent Predictors of LANSS Pain Score in Patients with Spinal Cord Injury (Multiple Linear Regression).

Independent Variable	B (Unstandardized)	Std. Error	β (Standardized)	*t*	*p*	VIF
VAS Paresthesia	1.100	0.187	0.605	5.877	<0.001	2.114
SCIM-III Score	−0.055	0.027	−0.306	−2.005	0.049	4.645
SF-36 General Health	0.070	0.027	0.339	2.617	0.011	3.347

Model Summary: R = 0.821, R^2^ = 0.674, Adjusted R^2^ = 0.584, F(18,65) = 7.46, *p* < 0.001. Notes: Only variables with *p* < 0.05 are presented. Dependent variable: LANSS Pain Score.

## Data Availability

The original contributions presented in the study are included in the article, further inquiries can be directed to the corresponding authors.

## Supplementary Materials

The following supporting information can be downloaded at: https://www.mdpi.com/article/10.3390/medicina61112047/s1, Table S1: The results of the multiple linear regression analysis, including all tested variables, are shown in Supplementary Table S1.
