# Supplementary material for: Evaluation of the Relationship Between Pain and Functional Status, Depression, Anxiety and Quality of Life in Patients with Spinal Cord Injury: Neuropathic Pain in Spinal Cord Injury
Source: Medicina (Kaunas). 2025 Nov 17;61(11):2047. doi: 10.3390/medicina61112047 (PMC12654325; doi:10.3390/medicina61112047)
Supplement: Supplementary file 1 [file medicina-61-02047-s001.zip › medicina-3948463-supplementary.pdf]

Table S1. Multiple Linear Regression Analysis of Factors Associated with LANSS Pain Score.

Predictor Variable	B (Unstandardized Coefficient)	SE (Standard Error)	β (Standardized Coefficient)	t	p-value
(Constant)	3.838	3.349	—	1.146	0.256
Pain duration (months)	0.043	0.050	0.502	0.871	0.387
Beck Anxiety score	0.011	0.055	0.024	0.209	0.835
SF-36 Physical Function	0.057	0.038	0.169	1.496	0.139
SF-36 Physical Role Limitation	0.022	0.016	0.234	1.415	0.162
SF-36 Bodily Pain	0.006	0.029	0.034	0.221	0.826
SF-36 General Health	**0.072**	0.027	**0.347**	**2.677**	**0.009**
SF-36 Vitality	0.024	0.026	0.136	0.919	0.362
SF-36 Social Functioning	0.010	0.018	0.071	0.546	0.587
SF-36 Emotional Role Limitation	0.016	0.017	0.172	0.985	0.328
SF-36 Mental Health	-0.032	0.027	-0.174	-1.159	0.251
Total SF-36 Score	-0.162	0.105	-0.745	-1.542	0.128
WISCI Level	0.147	0.103	0.237	1.427	0.158
SCIM III Score	-0.051	0.027	-0.286	-1.865	0.067
VAS Pain	0.250	0.207	0.127	1.211	0.230
VAS Fatigue	-0.165	0.194	-0.085	-0.852	0.398
VAS Paresthesia	**1.181**	0.188	**0.649**	**6.297**	**<0.001**
Age	0.011	0.031	0.033	0.340	0.735
BMI	0.049	0.089	0.049	0.547	0.586
Duration of Injury (months)	-0.033	0.049	-0.386	-0.674	0.503

Model summary: R = 0.822, $R^2 = 0.675$, Adjusted $R^2 = 0.579$,$F(19, 64) = 7.001$, $p < 0.001$,Durbin–Watson = 1.893. **Significant predictors:** SF-36 General Health ($p = 0.009$) and VAS Paresthesia ($p < 0.001$) were independently associated with higher LANSS pain scores.

Supplementary Table S1 presents the results of a multiple linear regression analysis performed to identify factors associated with neuropathic pain severity, as measured by the **Leeds Assessment of Neuropathic Symptoms and Signs (LANSS) pain score**, in patients with spinal cord injury (SCI). The model included clinical, psychological, and functional variables such as pain duration, Beck Anxiety scores, SF-36 subscale scores, functional independence (SCIM III), walking ability (WISCI level), visual analog scale (VAS) ratings, age, body mass index (BMI), and duration of injury.

The regression model was statistically significant (**$F(19, 64) = 7.001$, $p < 0.001$**) and explained **67.5% of the variance ($R^2 = 0.675$)** in LANSS scores, with an **adjusted R^2 of 0.579**. The **Durbin–Watson statistic (1.893)** indicated that the assumption of independence of residuals was met.
